# Supplementary material for: Isolation and characterization of broad-range Staphylococcus epidermidis sepunaviruses
Source: Microbiology (Reading). 2026 May 7;172(5):001693. doi: 10.1099/mic.0.001693 (PMC13152194; doi:10.1099/mic.0.001693)
Supplement: Uncited Supplementary Material 1. [file mic-172-01693-s001.pdf]

Table S1. Isolation and genome information available of the *Staphylococcus epidermidis* isolates used in this study.

Strain	GC (ST)	Total length (bp)	No. of contigs	n50 (bp)	Geography (Year of isolation)	Reference	Accession No.
DAR1104	5 (2)	2670770	3	2624715	New York, USA (2007)	https://doi.org/10.1016/j.meegid.2010.12.005; sequenced - this study	
DAR1153	5 (23)	2629733	5	2511077	New York, USA (2007)	https://doi.org/10.1016/j.meegid.2010.12.005; sequenced - this study	
DAR1267	5 (2)	3096419	26	607557	New York, USA (2007)	https://doi.org/10.1016/j.meegid.2010.12.005; sequenced - this study	
DAR1268	5 (23)	2508626	2	2490231	New York, USA (2007)	https://doi.org/10.1016/j.meegid.2010.12.005; sequenced - this study	
DAR1374	5 (23)	2516472	2	2480149	New York, USA (2007)	https://doi.org/10.1016/j.meegid.2010.12.005; sequenced - this study	
DAR1835	5 (23)	3084661	22	679976	New York, USA (2007)	https://doi.org/10.1016/j.meegid.2010.12.005; sequenced - this study	
DAR1888	5 (2)	2701792	4	2606977	New York, USA (2007)	https://doi.org/10.1016/j.meegid.2010.12.005; sequenced - this study	
DAR1907	5 (2)	2792663	4	2727549	New York, USA (2007)	https://doi.org/10.1016/j.meegid.2010.12.005	CP013943.1
Gre26	4 (230)	2537601	2	2512961	Greece (1998)	https://doi.org/10.1128/JB.01484-06; sequenced - this study	
99-1352	4 (766)	2524224	5	2440691	Umeå, Sweden (1999)		
NCIMB 9992	1 (14)	2277276	821	4287	Bedford, UK (1961)	https://doi.org/10.1099/00221287-30-3-409; sequenced - this study	
NCIMB 8558	6 (5)	2375307	170	29687	- (1944)	-	
NCIMB 11536	7 (1123)	2430632	292	15679	Leeds, UK (1976)	https://doi.org/10.1099/00222615-12-1-71; sequenced - this study	
17Y000025	6 (83)	2473762	201	26191	NUH, Nottingham, UK (2015)	This study	
17Y001110	6 (5)	2423493	100	46400	NUH, Nottingham, UK (2015)	This study	
17Y001219	5 (2)	2723703	160	34282	NUH, Nottingham, UK (2015)	This study	
17Y001272	6 (5)	2431753	317	14534	NUH, Nottingham, UK (2015)	This study	
17Y000448	5 (87)	2467827	189	24000	NUH, Nottingham, UK (2015)	This study	
17Y000396	6 (81)	2416333	131	39810	NUH, Nottingham, UK (2015)	This study	
17Y000349	6 (5)	2480028	226	23129	NUH, Nottingham, UK (2015)	This study	
17Y000075	4 (1130)	2445231	156	31198	NUH, Nottingham, UK (2015)	This study	
17Y000436	5 (23)	2451963	99	42489	NUH, Nottingham, UK (2015)	This study	
17Y001215	6 (83)	2490218	55	82468	NUH, Nottingham, UK (2015)	This study	
17Y000445	6 (307)	2562248	203	22925	NUH, Nottingham, UK (2015)	This study	
17Y000426	6 (59)	2483419	137	36036	NUH, Nottingham, UK (2015)	This study	
17Y000418	6 (210)	2446001	110	43720	NUH, Nottingham, UK (2015)	This study	
17Y000399	6 (5)	2462910	119	51594	NUH, Nottingham, UK (2015)	This study	
17Y000403	5 (2)	2594219	63	90350	NUH, Nottingham, UK (2015)	This study	
17Y000411	4 (203)	2466559	216	28920	NUH, Nottingham, UK (2015)	This study	
17Y000391	5 (87)	2506690	66	82787	NUH, Nottingham, UK (2015)	This study	
17Y001232	1 (1)	2336938	76	54226	NUH, Nottingham, UK (2015)	This study	
PS08	4 (1141)	2664905	3	2518368	Barcelona, Spain (2017)	https://doi.org/10.1016/j.jhin.2020.04.026; sequenced - https://doi.org/10.1101/2024.09.30.615834	CP170251.1
PS21	4 (1142)	2655004	3	2457908	Barcelona, Spain (2017)	https://doi.org/10.1016/j.jhin.2020.04.026; sequenced - https://doi.org/10.1101/2024.09.30.615834	CP170250.1
HS01	4 (332)	2516486	1	2516486	Barcelona, Spain (2013)	https://doi.org/10.1016/j.jhin.2020.04.026; sequenced - https://doi.org/10.1101/2024.09.30.615834	CP170249.1
PS25	4 (1006)	2618294	3	2438808	Barcelona, Spain (2017)	https://doi.org/10.1016/j.jhin.2020.04.026; sequenced - https://doi.org/10.1101/2024.09.30.615834	CP170248.1
Q10	4 (368)	2631764	3	2486279	Barcelona, Spain (2018)	https://doi.org/10.1016/j.jhin.2020.04.026; sequenced - https://doi.org/10.1101/2024.09.30.615834	CP170247.1
Q8	4 (1143)	2564838	4	1416651	Barcelona, Spain (2018)	https://doi.org/10.1016/j.jhin.2020.04.026; sequenced - https://doi.org/10.1101/2024.09.30.615834	CP170246.1
Q17	4 (1144)	2502292	1	2502292	Barcelona, Spain (2019)	https://doi.org/10.1016/j.jhin.2020.04.026; sequenced - https://doi.org/10.1101/2024.09.30.615834	CP170245.1
CCARM 3A673	4 (1148)	2752345	8	2585549	South Korea (2000)	https://doi.org/10.1089/mdr.2019.0151; sequenced - https://doi.org/10.1101/2024.09.30.615834	JBJHQI000000000.1
CCARM 3A593	4 (1094)	2604427	13	509713	South Korea (2015)	https://doi.org/10.1089/mdr.2019.0151; sequenced - https://doi.org/10.1101/2024.09.30.615834	JBICQX000000000.1
CCARM 3A686	4 (1145)	2807411	7	2599552	South Korea (2000)	https://doi.org/10.1089/mdr.2019.0151; sequenced - https://doi.org/10.1101/2024.09.30.615834	CP170661.1
CCARM 3A616	2 (763)	2497149	1	2497149	South Korea (2015)	https://doi.org/10.1089/mdr.2019.0151; sequenced - https://doi.org/10.1101/2024.09.30.615834	CP170244.1
CCARM 3A600	4 (1146)	2486232	2	2467735	South Korea (2015)	https://doi.org/10.1089/mdr.2019.0151; sequenced - https://doi.org/10.1101/2024.09.30.615834	CP170659.1
G11	6 (575)	2520397	32	226784	Scotland, UK (2021)	This study	
G13	6 (100)	2433237	27	235002	Scotland, UK (2021)	This study	

G14	6 (100)	2462552	29	147477	Scotland, UK (2021)	This study
G15	6 (99)	2519505	24	375849	Scotland, UK (2021)	This study
G26	6 (100)	2469612	32	184422	Scotland, UK (2021)	This study
G27	6 (99)	2498824	22	370899	Scotland, UK (2021)	This study
M39 22 1	6 (5)	-	-	-	England, UK (2013)	https://doi.org/10.1038/s41467-025-62584-2
P62C 50	6 (32)	-	-	-	England, UK (2013)	https://doi.org/10.1038/s41467-025-62584-2
P75F 1	4 (640)	-	-	-	England, UK (2013)	https://doi.org/10.1038/s41467-025-62584-2
M39 22 2	6 (5)	-	-	-	England, UK (2013)	https://doi.org/10.1038/s41467-025-62584-2
M39 8 10	- (Novel)	-	-	-	England, UK (2013)	https://doi.org/10.1038/s41467-025-62584-2
P62C B	6 (32)	-	-	-	England, UK (2013)	https://doi.org/10.1038/s41467-025-62584-2
P62C A	6 (32)	-	-	-	England, UK (2013)	https://doi.org/10.1038/s41467-025-62584-2
P62J 4	1 (73)	-	-	-	England, UK (2013)	https://doi.org/10.1038/s41467-025-62584-2
P62J 5	6 (487)	-	-	-	England, UK (2013)	https://doi.org/10.1038/s41467-025-62584-2
P62J A	6 (487)	-	-	-	England, UK (2013)	https://doi.org/10.1038/s41467-025-62584-2
P62J B	6 (487)	-	-	-	England, UK (2013)	https://doi.org/10.1038/s41467-025-62584-2
P75B 9	5 (2)	-	-	-	England, UK (2013)	https://doi.org/10.1038/s41467-025-62584-2
P75F5	4 (559)	-	-	-	England, UK (2013)	https://doi.org/10.1038/s41467-025-62584-2
P75F6	4 (640)	-	-	-	England, UK (2013)	https://doi.org/10.1038/s41467-025-62584-2
P75F7	4 (559)	-	-	-	England, UK (2013)	https://doi.org/10.1038/s41467-025-62584-2
P75F8	4 (559)	-	-	-	England, UK (2013)	https://doi.org/10.1038/s41467-025-62584-2

Table S2. Sequencing information available of the *Staphylococcus epidermidis* isolates used in this study.

Strain	Sequencing Method	Illumina Library Prep Kit	Nanopore ligation Kit	Guppy Version
99-1352	Hybrid	Nextera XT	SQK-LSK108	6.5.7
CCARM 3A593	Hybrid	Nextera XT	SQK-LSK109	3.6.1
CCARM 3A600	Hybrid	Nextera XT	SQK-LSK109	3.6.1
CCARM 3A616	Hybrid	Nextera XT	SQK-LSK109	3.6.1
CCARM 3A673	Hybrid	Nextera XT	SQK-LSK109	3.6.1
CCARM 3A686	Hybrid	Nextera XT	SQK-LSK109	3.6.1
DAR1104	Hybrid	Nextera XT	SQK-LSK108	6.5.7
DAR1153	Hybrid	Nextera XT	SQK-LSK108	6.5.7
DAR1267	Hybrid	Nextera XT	SQK-LSK108	6.5.7
DAR1268	Hybrid	Nextera XT	SQK-LSK108	6.5.7
DAR1374	Hybrid	Nextera XT	SQK-LSK108	6.5.7
DAR1835	Hybrid	Nextera XT	SQK-LSK108	6.5.7
DAR1888	Hybrid	Nextera XT	SQK-LSK108	6.5.7
HS01	Hybrid	Nextera XT	SQK-LSK109	3.6.1
PS08	Hybrid	Nextera XT	SQK-LSK109	3.6.1
PS21	Hybrid	Nextera XT	SQK-LSK109	3.6.1
PS25	Hybrid	Nextera XT	SQK-LSK109	3.6.1
Q10	Hybrid	Nextera XT	SQK-LSK109	3.6.1
Q17	Hybrid	Nextera XT	SQK-LSK109	3.6.1
Q8	Hybrid	Nextera XT	SQK-LSK109	3.6.1
17Y000025	Illumina	Nextera XT	N/A	N/A
17Y000075	Illumina	Nextera XT	N/A	N/A
17Y000349	Illumina	Nextera XT	N/A	N/A
17Y000391	Illumina	DNA Prep	N/A	N/A
17Y000396	Illumina	Nextera XT	N/A	N/A
17Y000399	Illumina	Nextera XT	N/A	N/A
17Y000403	Illumina	Nextera XT	N/A	N/A
17Y000411	Illumina	Nextera XT	N/A	N/A
17Y000418	Illumina	Nextera XT	N/A	N/A
17Y000426	Illumina	Nextera XT	N/A	N/A
17Y000436	Illumina	Nextera XT	N/A	N/A
17Y000445	Illumina	Nextera XT	N/A	N/A
17Y000448	Illumina	Nextera XT	N/A	N/A
17Y001110	Illumina	Nextera XT	N/A	N/A
17Y001215	Illumina	DNA Prep	N/A	N/A
17Y001219	Illumina	Nextera XT	N/A	N/A
17Y001232	Illumina	Nextera XT	N/A	N/A
17Y001272	Illumina	Nextera XT	N/A	N/A
G11	Illumina	Nextera XT	N/A	N/A
G13	Illumina	Nextera XT	N/A	N/A
G14	Illumina	Nextera XT	N/A	N/A
G15	Illumina	Nextera XT	N/A	N/A
G26	Illumina	Nextera XT	N/A	N/A
G27	Illumina	Nextera XT	N/A	N/A
NCIMB 11536	Illumina	Nextera XT	N/A	N/A
NCIMB 8558	Illumina	Nextera XT	N/A	N/A
NCIMB 9992	Illumina	Nextera XT	N/A	N/A
Gre26	Oxford Nanopore		SQK-LSK108	6.5.7
DAR1907	PacBio	N/A	N/A	N/A

Table S3. Prophages detected in the *S. epidermidis* isolates sequenced. The presence of superinfection exclusion and defense systems' genes is indicated.

Bacterial strain _Prophage	Contig	Location (Region length)	Completeness (score)	DF gene (DefenseFinder)	Superinfection exclussion Hit
ATCC 12228_1	Complete genome	40417-104206 (63.7 Kb)	intact (150)	AbiJ__AbiJ	
ATCC 12228_2	Complete genome	998407-1009443 (11 Kb)	incomplete (50)		1
DAR1104_1	1	1237811-1276423 (38.6 Kb)	intact (140)		
DAR1104_2	1	1862957-1904946 (41.9 Kb)	intact (150)		1
DAR1153_1	2	134-30145 (30 Kb)	intact (130)		1
DAR1153_2	2	536723-549212 (12.4 Kb)	incomplete (50)		1
DAR1153_3	1	221-42472 (42.2 Kb)	intact (150)		
DAR1267_1	12	17144-38604 (21.4 Kb)	incomplete (60)		
DAR1267_2	19	488678-529810 (41.1 Kb)	intact (150)		1
DAR1267_3	19	809583-851773 (42.1 Kb)	intact (140)		
DAR1267_4	21	517-42719 (42.2 Kb)	intact (150)		1
DAR1267_5	25	269-28270 (28 Kb)	incomplete (40)		1
DAR1267_6	25	56202-92361 (36.1 Kb)	intact (130)		
DAR1267_7	26	235-106261 (106 Kb)	intact (150)		
DAR1268_1	1	1687288-1701109 (13.8 Kb)	incomplete (50)		1
DAR1374_1	1	788805-799824 (11 Kb)	incomplete (50)		1
DAR1835_1	12	59-42249 (42.1 Kb)	intact (140)		
DAR1835_2	3	11421-76466 (65 Kb)	intact (150)		2
DAR1835_3	4	12394-48553 (36.1 Kb)	intact (130)		
DAR1835_4	6	779609-812651 (33 Kb)	intact (150)		1
DAR1835_5	8	11-139736 (139.7 Kb)	intact (150)		
DAR1835_6	9	77222-98682 (21.4 Kb)	incomplete (60)		
DAR1888_1	1	219561-258173 (38.6 Kb)	intact (140)		
DAR1888_2	1	844707-886696 (41.9 Kb)	intact (150)		1
DAR1907_1	Complete genome	612279-655144 (42.8 Kb)	intact (150)		
DAR1907_2	Complete genome	1524654-1560818 (36.1 Kb)	intact (130)		
DAR1907_3	Complete genome	1680737-1823843 (143.1 Kb)	intact (150)		
DAR1907_4	plasmid	68-22617 (22.5 Kb)	incomplete (60)		
G11_1	1	260572-303050 (42.4 Kb)	intact (150)		
G13_1	10	5346-70012 (64.6 Kb)	intact (150)	PD-Lambda-1 _ PD-Lambda-1	1
G14_1	9	81104-111236 (30.1 Kb)	intact (150)		
G14_2	13	111-12429 (12.3 Kb)	incomplete (20)	PD-Lambda-1 _ PD-Lambda-1	
G14_3	15	9570-44669 (35.1 Kb)	intact (150)		1
G15_1	1	65675-111138 (45.4 Kb)	intact (150)	PD-Lambda-1 _ PD-Lambda-1	
G15_2	2	199131-229184 (30 Kb)	questionable (70)		
G26_1	10	81104-111236 (30.1 Kb)	intact (150)		
G26_2	13	27740-52986 (25.2 Kb)	incomplete (20)	PD-Lambda-1 _ PD-Lambda-1	
G26_3	15	9570-44669 (35.1 Kb)	intact (150)		1
G27_1	2	199132-229185 (30 Kb)	questionable (70)		
G27_2	10	306-45745 (45.4 Kb)	intact (150)	PD-Lambda-1 _ PD-Lambda-1	
JCT0088_1	17	136-25833 (25.6 Kb)	questionable (80)		1
JCT0192_1	66	103-9700 (9.5 Kb)	questionable (80)		
JCT0194_1	1	22298-64488 (42.1 Kb)	intact (140)		
JCT0194_2	12	171-48094 (47.9 Kb)	questionable (70)		
JCT0194_3	51	644-19624 (18.9 Kb)	intact (100)		

JCT0194_4	71	2-12566 (12.5 Kb)	questionable (90)		
JCT0194_5	86	2446793 (8.5 Kb)	questionable (70)		
JCT0196_1	1	7818-65364 (57.5 Kb)	intact (150)		
JCT0200_1	31	1481-24740 (23.2 Kb)	intact (150)		
JCT0206_1	1	140-8100 (7.9 Kb)	incomplete (50)		1
JCT0207_1	1	155743-189796 (34 Kb)	intact (150)		1
JCT0209_1	1	8637-100669 (92 Kb)	intact (150)	RM_Type_IIG__Type_IIG_1, RM_Type_IIG__Type_IIG_1	
JCT0209_2	77	1-10070 (10 Kb)	intact (120)		
JCT0210_1	6	2998-12434 (9.4 Kb)	incomplete (50)		1
JCT0210_2	7	12421-70333 (57.9 Kb)	intact (150)		
JCT0210_3	8	2527-21897 (19.3 Kb)	incomplete (40)	AbiH__AbiH	
JCT0212_1	11	125-63784 (63.6 Kb)	intact (150)		
JCT0213_1	1	48278-79896 (31.6 Kb)	intact (100)	AbiQ__AbiQ	
JCT0213_2	1	1089994-1128399 (38.4 Kb)	intact (140)		
JCT0213_3	1	1715051-1757039 (41.9 Kb)	intact (150)		1
JCT0213_4	1	2036089-2078706 (42.6 Kb)	intact (150)		
JCT0215_1	17	5308-71234 (65.9 Kb)	intact (150)		
JCT0373_1	1	1585534-1629225 (43.6 Kb)	intact (150)		
JCT0374_1	1	74764-115421 (40.6 Kb)	incomplete (60)		
JCT0374_2	1	2026013-2042053 (16 Kb)	questionable (70)		1
JCT0376_1	3	194-34052 (33.8 Kb)	questionable (80)		
JCT0376_2	1	1698887-1741666 (42.7 Kb)	intact (150)		1
JCT0376_3	1	2289900-2331332 (41.4 Kb)	incomplete (60)		
JCT0376_4	1	2415210-2486277 (71 Kb)	intact (150)		
JCT0377_1	1	452027-465229 (13.2 Kb)	incomplete (50)		1
JCT0377_2	1	1101796-1146851 (45 Kb)	questionable (75)		
JCT0400_1	5	3-15151 (15.1 Kb)	questionable (80)		
JCT0400_2	3	40852-72878 (32 Kb)	questionable (90)	Bunzi__BnzB, Bunzi__BnzA, Stk2__Stk2	
JCT0400_3	1	431102-466040 (34.9 Kb)	intact (150)		1
JCT0400_4	1	2331853-2372515 (40.6 Kb)	questionable (70)	MazEF__MazF, MazEF__MazE, RM_Type_IIG__Type_IIG_FAM_1.einsi_trimmed	
JCT0400_5	1	2547229-2581759 (34.5 Kb)	questionable (70)		
JCT0401_1	12	6618-38170 (31.5 Kb)	questionable (80)		
JCT0401_2	3	264435-315265 (50.8 Kb)	intact (130)		1
JCT0402_1	1	2355746-2389006 (33.2 Kb)	intact (110)	MazEF__MazE, MazEF__MazF	
JCT0403_1	9	35541-68602 (33 Kb)	intact (100)	RM__Type_I_REases_FAM_0.einsi_trimmed, RM_Type_I_S_06, RM_Type_I_MTases_FAM_0	
JCT0403_2	9	2430374-2466451 (36 Kb)	questionable (75)		
ATCC 35984_1	Complete genome	891998-903017 (11 Kb)	incomplete (50)		1
ATCC 35984_2	Complete genome	1563904-1697887 (133.9 Kb)	intact (150)		
jct0047_1	1	95888-141889 (46 Kb)	incomplete (40)		
jct0047_2	1	787680-822618 (34.9 Kb)	intact (150)		1

Table S5. List of mutations detected and shared by Steph1, Steph3 and Steph4, but absent in Steph2.

Phage	Position	Type	Reference	Alternative	Strand	CDS	Product	Nucleotide position	Amino acid position	Effect
Steph1	183	snp	G	A	+	CDS_0001	hp	147/201	49/66	synonymous // Arg49Arg
Steph3	183	snp	G	A	+	CDS_0001	hp	147/201	49/66	synonymous // Arg49Arg
Steph4	183	snp	G	T	+	CDS_0001	hp	147/201	49/66	missense // Arg49Ser
Steph1	198	complex	ACTAATGT	GCTATTTC	+	CDS_0001	hp	162/201	54/66	missense // Met56Phe
Steph3	198	snp	A	G	+	CDS_0001	hp	162/201	54/66	synonymous // Glu54Glu
Steph4	198	complex	ACTAATGT	GCTATTTC	+	CDS_0001	hp	162/201	54/66	missense // Met56Phe
Steph1	2978	snp	A	G	+	CDS_0004	membrane protein	61/273	21/90	missense // Arg21Gly
Steph3	2978	snp	A	G	+	CDS_0004	membrane protein	61/273	21/90	missense // Arg21Gly
Steph4	2978	snp	A	G	+	CDS_0004	membrane protein	61/273	21/90	missense // Arg21Gly
Steph1	13749	snp	G	A	+	CDS_0030	hp	147/201	49/66	synonymous // Arg49Arg
Steph3	13749	snp	G	A	+	CDS_0030	hp	147/201	49/66	synonymous // Arg49Arg
Steph4	13749	snp	G	T	+	CDS_0030	hp	147/201	49/66	missense // Arg49Ser
Steph1	16651	snp	T	G	+	CDS_0038	hp	42/444	14/147	missense // Asn14Lys
Steph3	16651	snp	T	G	+	CDS_0038	hp	42/444	14/147	missense // Asn14Lys
Steph4	16651	snp	T	G	+	CDS_0038	hp	42/444	14/147	missense // Asn14Lys
Steph1	16671	snp	A	G	+	CDS_0038	hp	62/444	21/147	missense // Lys21Arg
Steph3	16671	snp	A	G	+	CDS_0038	hp	62/444	21/147	missense // Lys21Arg
Steph4	16671	snp	A	G	+	CDS_0038	hp	62/444	21/147	missense // Lys21Arg
Steph1	16808	snp	A	G	+	CDS_0038	hp	199/444	67/147	missense // Thr67Ala
Steph3	16808	snp	A	G	+	CDS_0038	hp	199/444	67/147	missense // Thr67Ala
Steph4	16808	snp	A	G	+	CDS_0038	hp	199/444	67/147	missense // Thr67Ala
Steph1	16908	snp	T	C	+	CDS_0038	hp	299/444	100/147	missense // Leu100Ser
Steph3	16908	snp	T	C	+	CDS_0038	hp	299/444	100/147	missense // Leu100Ser
Steph4	16908	snp	T	C	+	CDS_0038	hp	299/444	100/147	missense // Leu100Ser
Steph1	16981	snp	G	A	+	CDS_0038	hp	372/444	124/147	synonymous // Glu124Glu
Steph3	16981	snp	G	A	+	CDS_0038	hp	372/444	124/147	synonymous // Glu124Glu
Steph4	16981	snp	G	A	+	CDS_0038	hp	372/444	124/147	synonymous // Glu124Glu
Steph1	16988	snp	G	A	+	CDS_0038	hp	379/444	127/147	missense // Gly127Ser
Steph3	16988	snp	G	A	+	CDS_0038	hp	379/444	127/147	missense // Gly127Ser
Steph4	16988	snp	G	A	+	CDS_0038	hp	379/444	127/147	missense // Gly127Ser
Steph1	16999	snp	A	C	+	CDS_0038	hp	390/444	130/147	missense // Leu130Phe
Steph3	16999	snp	A	C	+	CDS_0038	hp	390/444	130/147	missense // Leu130Phe
Steph4	16999	snp	A	C	+	CDS_0038	hp	390/444	130/147	missense // Leu130Phe
Steph1	17032	snp	A	G	+	CDS_0038	hp	423/444	141/147	synonymous // Lys141Lys
Steph3	17032	snp	A	G	+	CDS_0038	hp	423/444	141/147	synonymous // Lys141Lys
Steph4	17032	snp	A	G	+	CDS_0038	hp	423/444	141/147	synonymous // Lys141Lys
Steph1	17063	snp	C	A		-				
Steph3	17063	snp	C	A		-				
Steph4	17063	snp	C	A		-				
Steph1	17081	snp	T	G	+	CDS_0039	hp	15/465	5/154	synonymous // Val5Val
Steph3	17081	snp	T	G	+	CDS_0039	hp	15/465	5/154	synonymous // Val5Val
Steph4	17081	snp	T	G	+	CDS_0039	hp	15/465	5/154	synonymous // Val5Val
Steph1	17087	snp	T	C	+	CDS_0039	hp	21/465	7/154	synonymous // Asp7Asp
Steph3	17087	snp	T	C	+	CDS_0039	hp	21/465	7/154	synonymous // Asp7Asp
Steph4	17087	snp	T	C	+	CDS_0039	hp	21/465	7/154	synonymous // Asp7Asp
Steph1	22765	snp	A	G	+	CDS_0049	hp	220/675	74/224	missense // Thr74Ala
Steph3	22765	snp	A	G	+	CDS_0049	hp	220/675	74/224	missense // Thr74Ala
Steph4	22765	snp	A	G	+	CDS_0049	hp	220/675	74/224	missense // Thr74Ala
Steph1	22930	snp	A	G	+	CDS_0049	hp	385/675	129/224	missense // Thr129Ala
Steph3	22930	snp	A	G	+	CDS_0049	hp	385/675	129/224	missense // Thr129Ala
Steph4	22930	snp	A	G	+	CDS_0049	hp	385/675	129/224	missense // Thr129Ala
Steph1	26461	snp	G	A	+	CDS_0059	hp	52/111	18/36	missense // Val18Ile
Steph3	26461	snp	G	A	+	CDS_0059	hp	52/111	18/36	missense // Val18Ile
Steph4	26461	snp	G	A	+	CDS_0059	hp	52/111	18/36	missense // Val18Ile
Steph1	26493	complex	CG	T	+	CDS_0059	hp	84/111	28/36	frameshift & synonymous // Glu29fs
Steph3	26493	complex	CG	T	+	CDS_0059	hp	84/111	28/36	frameshift & synonymous // Glu29fs
Steph4	26493	complex	CG	T	+	CDS_0059	hp	84/111	28/36	frameshift & synonymous // Glu29fs
Steph1	26502	snp	G	T	+	CDS_0059	hp	93/111	31/36	missense // Lys31Asn
Steph3	26502	snp	G	T	+	CDS_0059	hp	93/111	31/36	missense // Lys31Asn
Steph4	26502	snp	G	T	+	CDS_0059	hp	93/111	31/36	missense // Lys31Asn
Steph1	26514	snp	T	C	+	CDS_0059	hp	105/111	35/36	synonymous // Phe35Phe
Steph3	26514	snp	T	C	+	CDS_0059	hp	105/111	35/36	synonymous // Phe35Phe
Steph4	26514	snp	T	C	+	CDS_0059	hp	105/111	35/36	synonymous // Phe35Phe
Steph1	26548	snp	G	A		-				
Steph3	26548	snp	G	A		-				
Steph4	26548	snp	G	A		-				
Steph1	26970	del	GA	G	+	CDS_0061	hp	71/96	24/31	frameshift // Asn24fs
Steph3	26970	del	GA	G	+	CDS_0061	hp	71/96	24/31	frameshift // Asn24fs
Steph4	26970	del	GA	G	+	CDS_0061	hp	71/96	24/31	frameshift // Asn24fs
Steph1	28164	del	GGGGTTGT	T	+	CDS_0064	hp	53/105	18/34	frameshift // Asn18fs
Steph3	28164	del	GGGGTTGT	T	+	CDS_0064	hp	53/105	18/34	frameshift // Asn18fs
Steph4	28164	del	GGGGTTGT	T	+	CDS_0064	hp	53/105	18/34	frameshift // Asn18fs
Steph1	28595	complex	A	AATAAATAAAT		-				
Steph3	28595	complex	A	TAAATAAAT		-				
Steph4	28595	complex	A	TAAATAAATAAATAAA		-				
Steph1	28669	snp	C	T	+	CDS_0066	hp	43/570	15/189	synonymous // Leu15Leu
Steph3	28669	snp	C	T	+	CDS_0066	hp	43/570	15/189	synonymous // Leu15Leu
Steph4	28669	snp	C	T	+	CDS_0066	hp	43/570	15/189	synonymous // Leu15Leu
Steph1	28674	complex	CAG	TAA	+	CDS_0066	hp	48/570	16/189	missense // Arg17Lys
Steph3	28674	complex	CAG	TAA	+	CDS_0066	hp	48/570	16/189	missense // Arg17Lys
Steph4	28674	complex	CAG	TAA	+	CDS_0066	hp	48/570	16/189	missense // Arg17Lys
Steph1	28689	snp	C	T	+	CDS_0066	hp	63/570	21/189	synonymous // Asn21Asn
Steph3	28689	snp	C	T	+	CDS_0066	hp	63/570	21/189	synonymous // Asn21Asn
Steph4	28689	snp	C	T	+	CDS_0066	hp	63/570	21/189	synonymous // Asn21Asn
Steph1	28704	snp	G	A	+	CDS_0066	hp	78/570	26/189	synonymous // Val26Val
Steph3	28704	snp	G	A	+	CDS_0066	hp	78/570	26/189	synonymous // Val26Val
Steph4	28704	snp	G	A	+	CDS_0066	hp	78/570	26/189	synonymous // Val26Val
Steph1	28715	snp	A	G	+	CDS_0066	hp	89/570	30/189	missense // Asp30Gly
Steph3	28715	snp	A	G	+	CDS_0066	hp	89/570	30/189	missense // Asp30Gly
Steph4	28715	snp	A	G	+	CDS_0066	hp	89/570	30/189	missense // Asp30Gly
Steph1	28723	snp	G	A	+	CDS_0066	hp	97/570	33/189	missense // Asp33Asn
Steph3	28723	snp	G	A	+	CDS_0066	hp	97/570	33/189	missense // Asp33Asn
Steph4	28723	snp	G	A	+	CDS_0066	hp	97/570	33/189	missense // Asp33Asn
Steph1	28854	snp	C	A	+	CDS_0066	hp	228/570	76/189	missense // His76Gln
Steph3	28854	snp	C	A	+	CDS_0066	hp	228/570	76/189	missense // His76Gln
Steph4	28854	snp	C	A	+	CDS_0066	hp	228/570	76/189	missense // His76Gln
Steph1	28859	complex	AA	GC	+	CDS_0066	hp	233/570	78/189	missense // Glu78Gly
Steph3	28859	mnp	AA	GC	+	CDS_0066	hp	233/570	78/189	missense // Glu78Gly
Steph4	28859	snp	A	G	+	CDS_0066	hp	233/570	78/189	missense // Glu78Gly
Steph1	28896	snp	C	A	+	CDS_0066	hp	270/570	90/189	synonymous // Val90Val
Steph3	28896	snp	C	A	+	CDS_0066	hp	270/570	90/189	synonymous // Val90Val
Steph4	28896	snp	C	A	+	CDS_0066	hp	270/570	90/189	synonymous // Val90Val
Steph1	29055	snp	A	G	+	CDS_0066	hp	429/570	143/189	synonymous // Leu143Leu
Steph3	29055	snp	A	G	+	CDS_0066	hp	429/570	143/189	synonymous // Leu143Leu
Steph4	29055	snp	A	G	+	CDS_0066	hp	429/570	143/189	synonymous // Leu143Leu
Steph1	37189	snp	T	C	-	CDS_0088	transglycosylase	654/750	218/249	synonymous // Thr218Thr
Steph3	37189	snp	T	C	-	CDS_0088	transglycosylase	654/750	218/249	synonymous // Thr218Thr
Steph4	37189	snp	T	C	-	CDS_0088	transglycosylase	654/750	218/249	synonymous // Thr218Thr
Steph1	37197	complex	GCAATCCG	ACACGCCA	-	CDS_0088	transglycosylase	646/750	213/249	missense // Leu215Val
Steph3	37197	complex	GCAATCCG	ACACGCCA	-	CDS_0088	transglycosylase	646/750	213/249	missense // Leu215Val
Steph4	37200	complex	TCCGTGTGCGCCACGGG		-	CDS_0088	transglycosylase	643/750	211/249	missense // HisGlyLeu213ArgGlyVal
Steph1	42005	snp	T	C	-	CDS_0099	hp	214/261	72/86	missense // Ile72Val
Steph3	42005	snp	T	C	-	CDS_0099	hp	214/261	72/86	missense // Ile72Val
Steph4	42005	snp	T	C	-	CDS_0099	hp	214/261	72/86	missense // Ile72Val
Steph1	42048	complex	CACT	TACA	-	CDS_0099	hp	171/261	56/86	synonymous // 58
Steph3	42048	complex	CACT	TACA	-	CDS_0099	hp	171/261	56/86	synonymous // 58
Steph4	42048	complex	CACT	TACA	-	CDS_0099	hp	171/261	56/86	synonymous // 58
Steph1	42066	snp	T	C	-	CDS_0099	hp	153/261	51/86	synonymous // Leu51Leu

Steph3	42066	snp	T	C	-	CDS_0099	hp	153/261	51/86	synonymous // Leu51Leu
Steph4	42066	snp	T	C	-	CDS_0099	hp	153/261	51/86	synonymous // Leu51Leu
Steph1	42099	snp	A	G	-	CDS_0099	hp	120/261	40/86	synonymous // Phe40Phe
Steph3	42099	snp	A	G	-	CDS_0099	hp	120/261	40/86	synonymous // Phe40Phe
Steph4	42099	snp	A	G	-	CDS_0099	hp	120/261	40/86	synonymous // Phe40Phe
Steph1	42108	snp	A	T	-	CDS_0099	hp	111/261	37/86	synonymous // Ile37Ile
Steph3	42108	snp	A	T	-	CDS_0099	hp	111/261	37/86	synonymous // Ile37Ile
Steph4	42108	snp	A	T	-	CDS_0099	hp	111/261	37/86	synonymous // Ile37Ile
Steph1	42114	snp	T	C	-	CDS_0099	hp	105/261	35/86	synonymous // Glu35Glu
Steph3	42114	snp	T	C	-	CDS_0099	hp	105/261	35/86	synonymous // Glu35Glu
Steph4	42114	snp	T	C	-	CDS_0099	hp	105/261	35/86	synonymous // Glu35Glu
Steph1	42123	mnp	AT	GC	-	CDS_0099	hp	96/261	32/86	missense // Tyr32Cys
Steph3	42123	mnp	AT	GC	-	CDS_0099	hp	96/261	32/86	missense // Tyr32Cys
Steph4	42123	mnp	AT	GC	-	CDS_0099	hp	96/261	32/86	missense // Tyr32Cys
Steph1	42129	snp	G	A	-	CDS_0099	hp	90/261	30/86	synonymous // Gly30Gly
Steph3	42129	snp	G	A	-	CDS_0099	hp	90/261	30/86	synonymous // Gly30Gly
Steph4	42129	snp	G	A	-	CDS_0099	hp	90/261	30/86	synonymous // Gly30Gly
Steph1	42138	complex	CAA	TAG	-	CDS_0099	hp	81/261	27/86	synonymous // 28
Steph3	42138	complex	CAA	TAG	-	CDS_0099	hp	81/261	27/86	synonymous // 28
Steph4	42138	complex	CAA	TAG	-	CDS_0099	hp	81/261	27/86	synonymous // 28
Steph1	42250	snp	T	A	-	CDS_0100	DNA methyltransferase	903/930	301/309	missense // Glu301Asp
Steph3	42250	snp	T	A	-	CDS_0100	DNA methyltransferase	903/930	301/309	missense // Glu301Asp
Steph4	42250	snp	T	A	-	CDS_0100	DNA methyltransferase	903/930	301/309	missense // Glu301Asp
Steph1	42271	snp	C	T	-	CDS_0100	DNA methyltransferase	882/930	294/309	synonymous // Glu294Glu
Steph3	42271	snp	C	T	-	CDS_0100	DNA methyltransferase	882/930	294/309	synonymous // Glu294Glu
Steph4	42271	snp	C	T	-	CDS_0100	DNA methyltransferase	882/930	294/309	synonymous // Glu294Glu
Steph1	42279	complex	TCAAT	CTAAC	-	CDS_0100	DNA methyltransferase	874/930	290/309	missense // Asn292Asp
Steph3	42279	mnp	TC	CT	-	CDS_0100	DNA methyltransferase	874/930	291/309	missense // Asn292Asp
Steph4	42279	complex	TC	CT	-	CDS_0100	DNA methyltransferase	874/930	291/309	missense // Asn292Asp
Steph3	42292	complex	AATG	TATA	-	CDS_0100	DNA methyltransferase	861/930	286/309	synonymous // 288
Steph1	42295	snp	G	A	-	CDS_0100	DNA methyltransferase	858/930	286/309	synonymous // Tyr286Tyr
Steph4	42295	snp	G	A	-	CDS_0100	DNA methyltransferase	858/930	286/309	synonymous // Tyr286Tyr
Steph1	44484	snp	T	A	-	CDS_0102	DNA methyltransferase	18/777	6/258	synonymous // Ile6Ile
Steph3	44484	snp	T	A	-	CDS_0102	DNA methyltransferase	18/777	6/258	synonymous // Ile6Ile
Steph4	44484	snp	T	A	-	CDS_0102	DNA methyltransferase	18/777	6/258	synonymous // Ile6Ile
Steph1	44490	snp	G	A	-	CDS_0102	DNA methyltransferase	12/777	4/258	synonymous // Asn4Asn
Steph3	44490	snp	G	A	-	CDS_0102	DNA methyltransferase	12/777	4/258	synonymous // Asn4Asn
Steph4	44490	snp	G	A	-	CDS_0102	DNA methyltransferase	12/777	4/258	synonymous // Asn4Asn
Steph1	44495	snp	G	A	-	CDS_0102	DNA methyltransferase	7/777	3/258	synonymous // Leu3Leu
Steph3	44495	snp	G	A	-	CDS_0102	DNA methyltransferase	7/777	3/258	synonymous // Leu3Leu
Steph4	44495	snp	G	A	-	CDS_0102	DNA methyltransferase	7/777	3/258	synonymous // Leu3Leu
Steph1	55167	snp	C	T	-	CDS_0128	hp	161/324	54/107	missense // Ser54Asn
Steph3	55167	snp	C	T	-	CDS_0128	hp	161/324	54/107	missense // Ser54Asn
Steph4	55167	snp	C	T	-	CDS_0128	hp	161/324	54/107	missense // Ser54Asn
Steph1	81919	snp	C	A		-				
Steph3	81919	snp	C	A		-				
Steph4	81919	snp	C	A		-				
Steph1	93782	snp	A	G	+	CDS_0179	virion structural protein	1396/2655	466/884	missense // Lys466Glu
Steph3	93782	snp	A	G	+	CDS_0179	virion structural protein	1396/2655	466/884	missense // Lys466Glu
Steph4	93782	snp	A	G	+	CDS_0179	virion structural protein	1396/2655	466/884	missense // Lys466Glu
Steph1	105793	snp	C	T	+	CDS_0187	HTH DNA binding protein	852/1614	284/537	synonymous // Asn284Asn
Steph3	105793	snp	C	T	+	CDS_0187	HTH DNA binding protein	852/1614	284/537	synonymous // Asn284Asn
Steph4	105793	complex	CTAC	TTAT	+	CDS_0187	HTH DNA binding protein	852/1614	284/537	synonymous // 286
Steph1	116408	snp	T	C	+	CDS_0200	ribonucleotide reductase large subunit	566/2118	189/705	missense // Leu189Ser
Steph3	116408	snp	T	C	+	CDS_0200	ribonucleotide reductase large subunit	566/2118	189/705	missense // Leu189Ser
Steph4	116408	snp	T	C	+	CDS_0200	ribonucleotide reductase large subunit	566/2118	189/705	missense // Leu189Ser
Steph1	137513	snp	A	G	+	CDS_0228	hp	161/702	54/233	missense // Asn54Ser
Steph3	137513	snp	A	G	+	CDS_0228	hp	161/702	54/233	missense // Asn54Ser
Steph4	137513	snp	A	G	+	CDS_0228	hp	161/702	54/233	missense // Asn54Ser
Steph1	28773	complex	GTATATG	AATAAAA	+	CDS_0066	hp	147/570	49/189	missense // MetTyrMet49IleIleLys
Steph3	28773	complex	GTATATG	AATAAAA	+	CDS_0066	hp	147/570	49/189	missense // MetTyrMet49IleIleLys
Steph4	28774	complex	TAT	AGA	+	CDS_0066	hp	148/570	50/189	missense // Tyr50Arg
Steph1	28786	complex	GT	AG	+	CDS_0066	hp	160/570	54/189	missense // Val54Ser
Steph3	28786	complex	GT	AG	+	CDS_0066	hp	160/570	54/189	missense // Val54Ser
Steph4	28786	complex	GT	AG	+	CDS_0066	hp	160/570	54/189	missense // Val54Ser
Steph1	28797	complex	AT	GC	+	CDS_0066	hp	171/570	57/189	missense // Phe58Leu
Steph3	28797	complex	AT	GC	+	CDS_0066	hp	171/570	57/189	missense // Phe58Leu
Steph4	28797	complex	AT	GC	+	CDS_0066	hp	171/570	57/189	missense // Phe58Leu
Steph1	28804	snp	C	T	+	CDS_0066	hp	178/570	60/189	missense // Leu60Phe
Steph3	28804	snp	C	T	+	CDS_0066	hp	178/570	60/189	missense // Leu60Phe
Steph4	28804	snp	C	T	+	CDS_0066	hp	178/570	60/189	missense // Leu60Phe
Steph1	28817	complex	TAA	CAG	+	CDS_0066	hp	191/570	64/189	missense // IleAsn64ThrAsp
Steph3	28817	complex	TAA	CAG	+	CDS_0066	hp	191/570	64/189	missense // IleAsn64ThrAsp
Steph4	28817	complex	AAACTATTT	AGAATACCT	+	CDS_0066	hp	191/570	64/189	missense // IleAsn64ThrGlu
Steph1	28833	complex	TAATAATT	CAACGGAC	+	CDS_0066	hp	207/570	69/189	missense // Asn71Gly
Steph3	28833	complex	TAATAATT	CAACGGAC	+	CDS_0066	hp	207/570	69/189	missense // Asn71Gly
Steph4	28833	complex	TAATAAT	CAATGGG	+	CDS_0066	hp	207/570	69/189	missense // Asn71Gly
Steph1	28847	complex	TG	CA	+	CDS_0066	hp	221/570	74/189	missense // Met74Thr
Steph3	28847	complex	TG	CA	+	CDS_0066	hp	221/570	74/189	missense // Met74Thr
Steph4	28847	complex	TG	CA	+	CDS_0066	hp	221/570	74/189	missense // Met74Thr

hp: hypothetical protein

Table S6. Structural equivalences per region (aa <=> aa) between the MTases of the broad- range phages and that of Steph2.

Query				Subject		
Steph1	1	259	<=>	Steph1	1	259
Steph1	1	259	<=>	Steph4	15	273
	1	21	<=>		15	35
Steph1	27	154	<=>	Steph3	41	168
	157	259	<=>		169	271
	1	21	<=>		15	35
	24	27	<=>		36	39
	28	54	<=>		42	68
	55	66	<=>		77	88
	67	84	<=>		92	109
Steph1	87	104	<=>	Steph2	110	127
	105	**113**	<=>		129	137
	**140**	143	<=>		138	141
	144	147	<=>		143	146
	154	157	<=>		149	152
	160	188	<=>		153	181
	189	258	<=>		189	258
Steph3	1	271	<=>	Steph3	1	271
	1	36	<=>		1	36
Steph3	41	164	<=>	Steph4	41	164
	169	271	<=>		171	273
	15	35	<=>		1	21
Steph3	41	168	<=>	Steph1	27	154
	169	271	<=>		157	259
	1	35	<=>		1	35
	41	68	<=>		41	68
	69	80	<=>		77	88
	81	98	<=>		92	109
Steph3	101	118	<=>	Steph2	110	127
	119	**127**	<=>		129	137
	**154**	160	<=>		138	144
	171	200	<=>		152	181
	201	270	<=>		189	258
Steph4	1	273	<=>	Steph4	1	273
	1	36	<=>		1	36
Steph4	41	164	<=>	Steph3	41	164
	171	273	<=>		169	271
Steph4	15	273	<=>	Steph1	1	259
	1	36	<=>		1	36
	37	40	<=>		39	42
	43	68	<=>		43	68
	69	80	<=>		77	88
	81	98	<=>		92	109
Steph4	101	118	<=>	Steph2	110	127
	119	**127**	<=>		129	137
	**154**	159	<=>		138	143
	164	170	<=>		145	151
	173	202	<=>		152	181
	203	272	<=>		189	258

Query				Subject		
Steph2	1	258	<=>	Steph2	1	258
	15	35	<=>		1	21
	36	39	<=>		24	27
	42	68	<=>		28	54
	77	88	<=>		55	66
	92	109	<=>		67	84
Steph2	110	127	<=>	Steph1	87	104
	129	137	<=>		105	**113**
	138	141	<=>		**140**	143
	143	146	<=>		144	147
	149	152	<=>		154	157
	153	181	<=>		160	188
	189	258	<=>		189	258
	1	35	<=>		1	35
	41	68	<=>		41	68
	77	88	<=>		69	80
	92	109	<=>		81	98
Steph2	110	127	<=>	Steph3	101	118
	129	137	<=>		119	**127**
	138	144	<=>		**154**	160
	152	181	<=>		171	200
	189	258	<=>		201	270
	1	36	<=>		1	36
	39	42	<=>		37	40
	43	68	<=>		43	68
	77	88	<=>		69	80
	92	109	<=>		81	98
Steph2	110	127	<=>	Steph4	101	118
	129	137	<=>		119	**127**
	138	143	<=>		**154**	159
	145	151	<=>		164	170
	152	181	<=>		173	202
	189	258	<=>		203	272

Beginning and end of the structural loop in broad range phages

Homodimers percentage of Structural Identity

(DALI)

	Steph2	Steph1	Steph3	Steph4
Steph2	100	26	30	30
Steph1		100	88	96
Steph3			100	92
Steph4				100

Table S7. CDSs of phages Steph1-4 with a predicted function associated to structural, tail, or depolymerase activity.

Phages	CDS	Start	End	Sense	Function	Product	RBP detect v3	DePP	DepoScope	Phage DPO	Putative Depolymerase activity
Steph1	CDS_0082	34416	34694	-	tail	MTP					
Steph1	CDS_0086	35753	36094	-	tail	MTP					
Steph1	CDS_0096	40172	40366	-	head and packaging	VSP					
Steph1	CDS_0132	55895	56314	+	head and packaging	terminase small subunit					
Steph1	CDS_0133	56325	56540	+	head and packaging	terminase large subunit					
Steph1	CDS_0135	56945	57577	+	head and packaging	terminase large subunit					
Steph1	CDS_0136	57914	58006	+	head and packaging	terminase large subunit					
Steph1	CDS_0138	58273	59043	+	head and packaging	terminase large subunit					
Steph1	CDS_0139	59084	59860	+	head and packaging	VSP					
Steph1	CDS_0143	60957	61328	+	head and packaging	portal protein					
Steph1	CDS_0144	61312	63021	+	head and packaging	portal protein					
Steph1	CDS_0145	63196	63933	+	head and packaging	head maturation protease					
Steph1	CDS_0147	65076	66467	+	head and packaging	major head protein					
Steph1	CDS_0149	66843	67751	+	tail	TFP					
Steph1	CDS_0150	67780	68640	+	head and packaging	VSP					
Steph1	CDS_0152	69291	70148	+	unknown function	hp				89	
Steph1	CDS_0154	70364	72148	+	tail	tail sheath					
Steph1	CDS_0155	72208	72573	+	head and packaging	VSP					
Steph1	CDS_0163	75365	75658	+	head and packaging	VSP					
Steph1	CDS_0164	75784	76194	+	tail	tail assembly chaperone					
Steph1	CDS_0166	76790	80107	+	tail	tail associated lysin					
Steph1	CDS_0170	82349	84955	+	tail	tail protein with lysin activity		83			
Steph1	CDS_0171	84970	85878	+	tail	tail protein with lysin activity					
Steph1	CDS_0172	85881	88034	+	other	glycerophosphoryl diester phosphodiesterase	99.81	86		86	
Steph1	CDS_0174	88834	89631	+	head and packaging	VSP					
Steph1	CDS_0176	90155	90859	+	tail	baseplate protein					
Steph1	CDS_0177	90872	91918	+	tail	baseplate wedge subunit					
Steph1	CDS_0178	91935	94589	+	head and packaging	VSP		93		98	1
Steph1	CDS_0179	94713	95234	+	head and packaging	VSP					
Steph1	CDS_0180	95255	98710	+	unknown function	hp		97			
Steph1	CDS_0182	98930	100858	+	head and packaging	VSP		93		86	
Steph1	CDS_0183	100871	101263	+	tail	TFP					
Steph1	CDS_0184	101270	102640	+	tail	TFP	99.92		100	100	1
Steph1	CDS_0189	107916	108479	+	unknown function	hp				89	
Steph1	CDS_0194	112504	113559	+	DNA, RNA and nucleoti	DNA primase				85	
Steph1	CDS_0199	115391	117508	+	DNA, RNA and nucleoti	ribonucleotide reductase large subunit		86		92	
Steph1	CDS_0205	120426	123647	+	DNA, RNA and nucleoti	DNA polymerase		85			
Steph1	CDS_0215	129272	130012	+	unknown function	hp				88	
Steph1	CDS_0216	130015	131280	+	DNA, RNA and nucleoti	exonuclease				80	
Steph1	CDS_0221	133380	134225	+	unknown function	hp				83	
Steph1	CDS_0225	135897	136385	+	head and packaging	VSP					
Steph2	CDS_0083	35323	35601	-	tail	MTP					
Steph2	CDS_0087	36660	37001	-	tail	MTP					
Steph2	CDS_0095	40095	40289	-	head and packaging	VSP					
Steph2	CDS_0132	56346	56765	+	head and packaging	terminase small subunit					
Steph2	CDS_0133	56776	56991	+	head and packaging	terminase large subunit					
Steph2	CDS_0135	57396	58028	+	head and packaging	terminase large subunit					
Steph2	CDS_0136	58365	58457	+	head and packaging	terminase large subunit					
Steph2	CDS_0138	58724	59494	+	head and packaging	terminase large subunit					
Steph2	CDS_0139	59535	60311	+	head and packaging	VSP					
Steph2	CDS_0143	61408	61779	+	head and packaging	portal protein					
Steph2	CDS_0144	61763	63472	+	head and packaging	portal protein					
Steph2	CDS_0145	63647	64384	+	head and packaging	head maturation protease					
Steph2	CDS_0147	65527	66918	+	head and packaging	major head protein					
Steph2	CDS_0149	67294	68202	+	tail	TFP					
Steph2	CDS_0150	68231	69091	+	head and packaging	VSP					
Steph2	CDS_0152	69742	70599	+	unknown function	hp				89	
Steph2	CDS_0154	70815	72599	+	tail	tail sheath					
Steph2	CDS_0155	72659	73024	+	head and packaging	VSP					
Steph2	CDS_0164	75817	76110	+	head and packaging	VSP					
Steph2	CDS_0165	76236	76646	+	tail	tail assembly chaperone					
Steph2	CDS_0167	77242	80559	+	tail	tail associated lysin					
Steph2	CDS_0171	82801	85407	+	tail	tail protein with lysin activity		83			
Steph2	CDS_0172	85422	86330	+	tail	tail protein with lysin activity					
Steph2	CDS_0173	86333	88486	+	other	glycerophosphoryl diester phosphodiesterase	99.81	86		86	
Steph2	CDS_0175	89286	90083	+	head and packaging	VSP					
Steph2	CDS_0177	90607	91311	+	tail	baseplate protein					
Steph2	CDS_0178	91324	92370	+	tail	baseplate wedge subunit					
Steph2	CDS_0179	92387	95041	+	head and packaging	VSP		93		98	1
Steph2	CDS_0180	95165	95686	+	head and packaging	VSP					
Steph2	CDS_0181	95707	99162	+	unknown function	hp		97			
Steph2	CDS_0183	99382	101310	+	head and packaging	VSP		93		86	
Steph2	CDS_0184	101323	101715	+	tail	TFP					
Steph2	CDS_0185	101722	103092	+	tail	TFP	99.92		100	100	1
Steph2	CDS_0190	108368	108931	+	unknown function	hp				89	
Steph2	CDS_0195	112956	114011	+	DNA, RNA and nucleoti	DNA primase				84	
Steph2	CDS_0200	115843	117960	+	DNA, RNA and nucleoti	ribonucleotide reductase large subunit		86		91	
Steph2	CDS_0206	120878	124099	+	DNA, RNA and nucleoti	DNA polymerase		85			
Steph2	CDS_0216	129724	130464	+	unknown function	hp				86	
Steph2	CDS_0217	130467	131732	+	DNA, RNA and nucleoti	exonuclease				80	
Steph2	CDS_0222	133832	134677	+	unknown function	hp				83	
Steph2	CDS_0226	136349	136837	+	head and packaging	VSP					
Steph3	CDS_0028	13176	13430	-	unknown function	hp			89		
Steph3	CDS_0067	28455	28733	-	tail	MTP					
Steph3	CDS_0071	29801	30142	-	tail	MTP					
Steph3	CDS_0080	33557	33802	-	head and packaging	VSP					
Steph3	CDS_0097	42090	43544	-	lysis	endolysin		83			
Steph3	CDS_0116	49324	49743	+	head and packaging	terminase small subunit					
Steph3	CDS_0117	49754	49969	+	head and packaging	terminase large subunit					
Steph3	CDS_0119	50374	51006	+	head and packaging	terminase large subunit					
Steph3	CDS_0120	51343	51435	+	head and packaging	terminase large subunit					
Steph3	CDS_0122	51702	52472	+	head and packaging	terminase large subunit					
Steph3	CDS_0123	52513	53289	+	head and packaging	VSP					
Steph3	CDS_0127	54386	54757	+	head and packaging	portal protein					
Steph3	CDS_0128	54741	56450	+	head and packaging	portal protein					
Steph3	CDS_0129	56601	57362	+	head and packaging	head maturation protease					
Steph3	CDS_0131	58514	59905	+	head and packaging	major head protein					
Steph3	CDS_0133	60281	61189	+	tail	TFP					
Steph3	CDS_0134	61218	62078	+	head and packaging	VSP					
Steph3	CDS_0136	62729	63586	+	unknown function	hp				86	
Steph3	CDS_0138	63802	65586	+	tail	tail sheath					

Steph3	CDS_0139	65646	66011	+	head and packaging	VSP					
Steph3	CDS_0148	68804	69097	+	head and packaging	VSP					
Steph3	CDS_0149	69223	69633	+	tail	tail assembly chaperone					
Steph3	CDS_0151	70229	73546	+	tail	tail associated lysin					
Steph3	CDS_0155	75788	78394	+	tail	tail protein with lysin activity		84			
Steph3	CDS_0156	78409	79317	+	tail	tail protein with lysin activity					
Steph3	CDS_0157	79320	81473	+	other	glycerophosphoryl diester phosphodiesterase	99.81	85		85	
Steph3	CDS_0160	82276	83073	+	head and packaging	VSP					
Steph3	CDS_0162	83597	84301	+	tail	baseplate protein					
Steph3	CDS_0163	84314	85360	+	tail	baseplate wedge subunit					
Steph3	CDS_0164	85377	88031	+	head and packaging	VSP		93		98	1
Steph3	CDS_0165	88155	88676	+	head and packaging	VSP					
Steph3	CDS_0166	88697	92152	+	unknown function	hp		97			
Steph3	CDS_0168	92372	94300	+	head and packaging	VSP		92		81	
Steph3	CDS_0169	94315	94707	+	tail	TFP					
Steph3	CDS_0170	94714	96084	+	tail	TFP	99.92		100	100	1
Steph3	CDS_0171	96176	99178	+	DNA, RNA and nucleoti	DNA helicase		90			
Steph3	CDS_0177	103930	104493	+	unknown function	hp				83	
Steph3	CDS_0182	108518	109573	+	DNA, RNA and nucleoti	DNA primase				84	
Steph3	CDS_0187	111405	112559	+	DNA, RNA and nucleoti	ribonucleotide reductase large subunit				82	
Steph3	CDS_0211	127334	128074	+	unknown function	hp				86	
Steph3	CDS_0217	131442	132287	+	unknown function	hp				86	
Steph3	CDS_0221	133959	134447	+	head and packaging	VSP					
Steph4	CDS_0078	33300	33578	-	tail	MTP					
Steph4	CDS_0082	34637	34978	-	tail	MTP					
Steph4	CDS_0090	37464	37658	-	head and packaging	VSP					
Steph4	CDS_0105	44661	46115	-	lysis	endolysin		82			
Steph4	CDS_0122	51882	52301	+	head and packaging	terminase small subunit					
Steph4	CDS_0123	52312	52527	+	head and packaging	terminase large subunit					
Steph4	CDS_0125	52932	53564	+	head and packaging	terminase large subunit					
Steph4	CDS_0126	53901	53993	+	head and packaging	terminase large subunit					
Steph4	CDS_0128	54260	55030	+	head and packaging	terminase large subunit					
Steph4	CDS_0129	55071	55847	+	head and packaging	VSP					
Steph4	CDS_0133	56944	57315	+	head and packaging	portal protein					
Steph4	CDS_0134	57299	59008	+	head and packaging	portal protein					
Steph4	CDS_0135	59183	59920	+	head and packaging	head maturation protease					
Steph4	CDS_0137	61063	62454	+	head and packaging	major head protein					
Steph4	CDS_0139	62830	63738	+	tail	TFP					
Steph4	CDS_0140	63767	64627	+	head and packaging	VSP					
Steph4	CDS_0142	65278	66135	+	unknown function	hp				89	
Steph4	CDS_0144	66351	68135	+	tail	tail sheath					
Steph4	CDS_0145	68195	68560	+	head and packaging	VSP					
Steph4	CDS_0153	71352	71645	+	head and packaging	VSP					
Steph4	CDS_0154	71771	72181	+	tail	tail assembly chaperone					
Steph4	CDS_0156	72777	76094	+	tail	tail associated lysin					
Steph4	CDS_0160	78336	80942	+	tail	tail protein with lysin activity		83			
Steph4	CDS_0161	80957	81865	+	tail	tail protein with lysin activity					
Steph4	CDS_0162	81868	84021	+	other	glycerophosphoryl diester phosphodiesterase	99.81	86		87	
Steph4	CDS_0164	84821	85618	+	head and packaging	VSP					
Steph4	CDS_0166	86142	86846	+	tail	baseplate protein					
Steph4	CDS_0167	86859	87905	+	tail	baseplate wedge subunit					
Steph4	CDS_0168	87922	90576	+	head and packaging	VSP		93		98	1
Steph4	CDS_0169	90700	91221	+	head and packaging	VSP					
Steph4	CDS_0170	91242	94697	+	unknown function	hp		97			
Steph4	CDS_0172	94917	96845	+	head and packaging	VSP		93		86	
Steph4	CDS_0173	96858	97250	+	tail	TFP					
Steph4	CDS_0174	97257	98627	+	tail	TFP	99.91		100	100	1
Steph4	CDS_0175	98719	101715	+	DNA, RNA and nucleoti	DNA helicase		88			
Steph4	CDS_0179	105157	105720	+	unknown function	hp				89	
Steph4	CDS_0184	109745	110800	+	DNA, RNA and nucleoti	DNA primase				85	
Steph4	CDS_0189	112635	114752	+	DNA, RNA and nucleoti	ribonucleotide reductase large subunit		86		91	
Steph4	CDS_0212	128986	129726	+	unknown function	hp				86	
Steph4	CDS_0213	129729	130994	+	DNA, RNA and nucleoti	exonuclease				80	
Steph4	CDS_0218	133094	133939	+	unknown function	hp				83	
Steph4	CDS_0222	135611	136099	+	head and packaging	VSP					

VSP: Virion structural protein, MTP: Major tail protein, TFP: Tail fiber protein, hp: hypothetical protein

This summary includes all CDSs annotated with a structural function (tail, head and packaging tail) predicted RBPs and CDSs with potential depolymerase activity.

	VSP	TFP	MTP	RBPs	Depos
Steph1	10	3	2	2	2
Steph2	10	3	2	2	2
Steph3	10	3	2	2	2
Steph4	10	3	2	2	2

Table S8. Differential probability (dPR) for the correlation against phage infection. Presence of defense systems' genes, genes of the *ica* operon and the presence of at least one intact prophage are evaluated for the infection pattern of each phage.

dPR: -1 to 1

			PADLOC										DefenseFinder				
	ica Operon	Prophages	cas_type_III-A	PDC-S06	ppl	Uzume	PDC-S61	RM_type_I	RM_type_IV	PD-T4-6	AbiD	PDC-S07	cas_type_III-A	RloC	RM_type_I	Abi2	AbiQ
Category	Others	Others	CrisprCas systems	Phage defense candidates	Others	Others	Phage defense candidates	Restriction modification	Restriction modification	Phage defense candidates	Abortive infection	Phage defense candidates	CrisprCas systems	Others	Restriction modification	Abortive infection	Abortive infection
Steph1	-0.590	-0.389	-0.350	-0.350	-0.278	-0.189	-0.156	-0.143	-0.074	-0.014	-0.125	0.085	-0.222	-0.222	-0.083	-0.014	0.051
Steph2	-0.267	-0.222	-0.200	-0.200	-0.111	-0.123	-0.063	-0.070	-0.042	-0.057	-0.031	0.205	-0.205	-0.205	-0.083	-0.057	0.068
Steph3	-0.590	-0.361	-0.375	-0.375	-0.306	-0.218	-0.188	-0.183	-0.037	0.021	-0.188	-0.026	-0.248	-0.248	-0.125	0.021	0.026
Steph4	-0.590	-0.389	-0.350	-0.350	-0.278	-0.189	-0.156	-0.143	-0.074	-0.014	-0.219	-0.051	-0.222	-0.222	-0.083	-0.014	0.051

Others · CrisprCas systems · Phage defense candidates · Restriction modification · Abortive infection

Table S10. Genome-wide association study (GWAS) of bacterial genes and phage resistance.

Phage	Gene	Annotation	Bonferroni_p
vb_sep_Steph1<br>vb_sep_Steph3<br>vb_sep_Steph4	*icaA*	poly-beta-16-N-acetyl-D-glucosamine synthase	2.97E-04
vb_sep_Steph1<br>vb_sep_Steph3<br>vb_sep_Steph4	*icaB*	intercellular adhesin biosynthesis polysaccharide N-deacetylase	2.97E-04
vb_sep_Steph1<br>vb_sep_Steph3<br>vb_sep_Steph4	*icaC*	polysaccharide intercellular adhesin biosynthesis/export protein IcaC	2.97E-04
vb_sep_Steph1<br>vb_sep_Steph3<br>vb_sep_Steph4	*icaD*	intracellular adhesion protein IcaD	2.97E-04
vb_sep_Steph1<br>vb_sep_Steph3<br>vb_sep_Steph4	*icaR*	Biofilm operon icaADBC HTH-type negative transcriptional regulator IcaR	2.97E-04
vb_sep_Steph1<br>vb_sep_Steph3<br>vb_sep_Steph4	group_3179 (Chromosome segregation ATPase Smc system + Nuclease SbcCD subunit C)	Chromosome segregation ATPase Smc; ATP-binding protein; AAA family ATPase; Nuclease SbcCD subunit C	3.07E-03
